# Adrenergic Mechanisms of Audiogenic Seizure-Induced Death in a Mouse Model of *SCN8A* Encephalopathy

**DOI:** 10.3389/fnins.2021.581048

**Published:** 2021-03-04

**Authors:** Eric R. Wengert, Ian C. Wenker, Elizabeth L. Wagner, Pravin K. Wagley, Ronald P. Gaykema, Jung-Bum Shin, Manoj K. Patel

**Affiliations:** ^1^Department of Anesthesiology, University of Virginia Health System, Charlottesville, VA, United States; ^2^Neuroscience Graduate Program, University of Virginia Health System, Charlottesville, VA, United States; ^3^Department of Neuroscience, University of Virginia School of Medicine, Charlottesville, VA, United States; ^4^Department of Biochemistry and Molecular Genetics, University of Virginia School of Medicine, Charlottesville, VA, United States

**Keywords:** SUDEP, *SCN8A* encephalopathy, respiratory arrest, adrenergic receptors, audiogenic seizures

## Abstract

Sudden unexpected death in epilepsy (SUDEP) is the leading cause of death amongst patients whose seizures are not adequately controlled by current therapies. Patients with *SCN8A* encephalopathy have an elevated risk for SUDEP. While transgenic mouse models have provided insight into the molecular mechanisms of *SCN8A* encephalopathy etiology, our understanding of seizure-induced death has been hampered by the inability to reliably trigger both seizures and seizure-induced death in these mice. Here, we demonstrate that mice harboring an *Scn8a* allele with the patient-derived mutation N1768D (D/+) are susceptible to audiogenic seizures and seizure-induced death. In adult D/+ mice, audiogenic seizures are non-fatal and have nearly identical behavioral, electrographical, and cardiorespiratory characteristics as spontaneous seizures. In contrast, at postnatal days 20–21, D/+ mice exhibit the same seizure behavior, but have a significantly higher incidence of seizure-induced death following an audiogenic seizure. Seizure-induced death was prevented by either stimulating breathing via mechanical ventilation or by acute activation of adrenergic receptors. Conversely, in adult D/+ mice inhibition of adrenergic receptors converted normally non-fatal audiogenic seizures into fatal seizures. Taken together, our studies show that in our novel audiogenic seizure-induced death model adrenergic receptor activation is necessary and sufficient for recovery of breathing and prevention of seizure-induced death.

## Introduction

Sudden unexpected death in epilepsy (SUDEP) is defined as the sudden, unexpected, non-traumatic, and non-drowning death of a person with epilepsy for which postmortem examination does not reveal an anatomical or pathological cause of death (Nashef et al., 2012). SUDEP is the most common cause of death associated with epilepsy, accounting for between 8 and 17% of all epilepsy-related deaths (Terra et al., 2013), and rising to 50% for patients with poorly controlled seizures (Devinsky, 2011; Terra et al., 2013). SUDEP primarily affects the young; among neurological conditions it takes a close second only to stroke for number of life-years lost (Devinsky et al., 2016).

Due to the unpredictable nature for instances of SUDEP, clinical mechanisms of death remain elusive. To circumvent this issue, several rodent models have been developed in recent years to gain mechanistic insights into SUDEP. Most clinical SUDEP cases are believed to occur after generalized tonic-clonic seizures (Dasheiff and Dickinson, 1986; Bird et al., 1997; Nilsson et al., 1999; So et al., 2000; Ryvlin et al., 2013). Thus, animal models in which death occurs immediately after convulsive seizures are used to study SUDEP. Such models include the DBA/1&2, Cacna1a^*S*218L^, Scn1a^*R*1407X^, RyR2^*R*176Q^, Scn1a KO, and Kcna1 KO mouse models as well as inducible kainic acid and maximal electroshock seizure models. These approaches have fueled various hypotheses concerning the mechanisms of SUDEP, including brainstem spreading depolarization (Aiba and Noebels, 2015; Aiba et al., 2016; Jansen et al., 2019; Loonen et al., 2019), autonomic dysregulation and cardiac arrhythmias (Glasscock et al., 2010; Auerbach et al., 2013; Kalume et al., 2013), and respiratory arrest due to central (Faingold et al., 2010; Buchanan et al., 2014; Kim et al., 2018; Kruse et al., 2019), or obstructive apnea (Nakase et al., 2016; Villiere et al., 2017; Irizarry et al., 2020). Of these models, only the Dravet Syndrome models (i.e., *Scn1a*^*R*1407X^ and *Scn1a KO*) directly represent a patient population that is susceptible to SUDEP (Escayg and Goldin, 2010; Kim et al., 2018). The genetic etiologies of DBA/1&2, *Cacna1a*^*S*218L^, *RyR2*^*R*176Q^, *Lmx1b*^*f/f/p*^, and *Kcna1 KO* mice are either unknown, identified from non-epilepsy patient populations, or are manipulations that lead to loss of an entire cell population, receptor subtypes, or ion channels, which are not known to occur in epilepsy patients.

In this study we used a transgenic mouse model of *SCN8A* epileptic encephalopathy, also known as Early Infantile Epileptic Encephalopathy 13 (EIEE13) (OMIM #614558). Patients with this recently recognized syndrome have *de novo*, gain-of-function *SCN8A* mutations and a high incidence of SUDEP ranging between 5 and 10%, although this is possibly an underestimate due to the young average patient age (Hammer et al., 1993; Ohba et al., 2014; Blanchard et al., 2015; Larsen et al., 2015; Gardella et al., 2018; Johannesen et al., 2018). This *Scn8a* mouse model harbors the germline knock-in mutation N1768D (D/+), a mutation identified in an *SCN8A* epilepsy patient that died of SUDEP (Wagnon et al., 2015; Lopez-Santiago et al., 2017; Ottolini et al., 2017; Sprissler et al., 2017; Wengert et al., 2019). The D/+ mice recapitulate the key hallmarks of *SCN8A* encephalopathy and SUDEP: chronic spontaneous tonic-clonic seizures and seizure-induced deaths (Wagnon et al., 2015). While the D/+ mice have been used to unravel mechanisms of disease etiology (Wagnon et al., 2015; Lopez-Santiago et al., 2017; Ottolini et al., 2017; Sprissler et al., 2017; Wengert et al., 2019), their utility as a SUDEP model has been hampered by the inability to temporally control the occurrence of seizures and seizure-induced death.

Here, we demonstrate the ability to evoke seizures on command in D/+ mice using high-intensity sound. These evoked seizures are nearly identical to spontaneous seizures with respect to behavioral, electrographic, and cardiorespiratory parameters. We also serendipitously determined a developmental window where audiogenic seizures almost always (∼85%) lead to seizure-induced death. Using our audiogenic seizure model, we demonstrate that (1) the primary cause of seizure-induced death is respiratory arrest that is initiated during the tonic phase, (2) non-fatal seizures also present with transient apnea but breathing recovers after the tonic phase, (3) peri-ictal adrenergic receptor activity is both necessary and sufficient for survival after a tonic-clonic seizure, and (4) the mechanism of action for adrenergic stimulation is breathing recovery after initial tonic phase apnea.

## Materials and Methods

### Mice

All mice were housed and maintained in accordance with the Animal Care and Use Committee standards of the University of Virginia in a temperature and humidity-controlled vivarium with a standard 12 h light/dark cycle with food and water *ad libitum*. Both male and female mice were used in roughly equal numbers, and no sex differences were observed for any of the experiments based on seizure behavior or risk of sudden death.

### Genotyping

Genotyping of transgenic mice was done using standard PCR techniques with DNA acquired from tail biopsies. Genotyping of D/+ mice was performed as previously described (Wagnon et al., 2015), using the primers 5′-TGACT GCAGC TTGGA CAAGG AGC-3′ and 5′-TCGATGGTGT TGGGC TTGGG TAC-3′. The resulting PCR product, a 327 bp genomic fragment of *SCN8A* containing the mutation, was then digested with *Hin*cII which generates a single fragment of 327 bp for the wild type allele and two fragments of 209 and 118 bp for the mutant allele.

### Audiogenic Seizure Assessment

To test for audiogenic seizures mice were taken from their home cage and transferred to a clean test cage where they were allowed to acclimate for ∼20 s before the onset of the acoustic stimulus. Similar to a method described previously (Martin et al., 2020), a sonicator (Branson 200 ultrasonic cleaner) was used to produce the audiogenic stimulus directly adjacent to the test cage. The stimulus duration lasted for 50 s or until the animal had a behavioral seizure.

Audiogenic seizures were recorded using a laptop webcam. Duration of seizure phases were analyzed by taking the time in seconds that the mouse spent in each of the phases: a wild-running phase characterized by fast circular running throughout the cage, a tonic phase characterized by hindlimb extension and muscle rigidity, a clonic phase typified by myoclonic jerking of the hindlimbs, and recovery exemplified when the mouse ceased myoclonic jerking and righted itself. In cases where death occurred, the end of the tonic phase was apparent at the point of hindlimb muscle relaxation. For all experiments involving rescue of seizure-induced sudden death, at least one control mouse from the experimental litter was confirmed to experience seizure-induced sudden death before conducting any rescue experiments on remaining littermates.

### Intensity-Dependence of Audiogenic Seizures

Intensity-dependent sensitivity of the audiogenic seizures was tested using adult D/+, placed in a custom wooden chamber and exposed to ∼20 s of pure tone acoustic stimulation (Audacity) at 14 kHz using a JBL speaker (Model #2446H JBL Incorporated). Tones were delivered beginning at 50 dB and manually increased in increments of ∼5 dB until the D/+ mouse exhibited an audiogenic seizure.

### Auditory Brainstem Responses

Auditory Brainstem Responses (ABRs) were recorded from WT and D/+ mice at P56 and P112 in a blinded manner, as previously described (Ruhl et al., 2019). The mice were anesthetized with a single intraperitoneal injection of 100 mg/kg ketamine hydrochloride (Fort Dodge Animal Health) and 10 mg/kg xylazine hydrochloride (Lloyd Laboratories). Body temperature was maintained with a Deltaphase isothermal heating pad (Braintree Scientific) during the procedure. The ABRs were performed in a sound-attenuating booth (Med-Associates) using equipment and software (Smart-EP) from Intelligent Hearing Systems. Recordings were collected through subdermal needle electrodes (Intelligent Hearing Systems). A non-inverting electrode was placed at the vertex of the midline, an inverting electrode was placed over the mastoid of the right ear, and a ground electrode was placed on the upper thigh. Pure tone stimuli of 31.3 μs were presented at the rate of 21.1/s through a high frequency transducer (Intelligent Hearing Systems). Responses were filtered at 300–3,000 Hz and threshold levels were determined from averages of 1,024 stimulus presentations. Stimulus intensity at each tested frequency (8, 11.3, 16, 22.4, and 32 kHz) was decreased from the maximum intensity in 5–10 dB steps until a waveform response could no longer be identified. The maximum intensities used were 120, 120, 110, 100, and 90 dB for 8, 11.3, 16, 22.4, and 32 kHz, respectively. The lowest intensity at which a response was observed was recorded as the threshold. If a waveform could not be identified at the maximum output of the transducer, a value of 5 dB was added to the maximum output as the threshold.

### Surgical Preparation

Custom ECoG/ECG headsets (PlasticsOne, Inc., or Pinnacle Technology Inc.) were implanted in 6–10-weeks-old D/+ mice using standard aseptic surgical techniques. Anesthesia was induced with 5% and maintained with 0.5–3% isoflurane. Adequacy of anesthesia was assessed by lack of toe-pinch reflex. A midline skin incision was made over the skull, and burr holes were made at the lateral/rostral end of both the left and right parietal bones to place ECoG leads, and at the interparietal bone for a reference and ground electrodes. Two ECG leads were passed subcutaneously to the left abdomen and right shoulder and sutured into place to approximate a lead II arrangement. The headsets were attached to the skull with dental acrylic (Jet Acrylic; Lang Dental). Mice received postoperative analgesia with meloxicam (0.5–1 mg/kg, i.p.) or ketoprofen (5 mg/kg, i.p.) and 0.9% saline (0.5 ml i.p.) and were allowed to recover a minimum of 2–5 days prior to experiments.

### Recording of ECoG, ECG, and Breathing for Spontaneous Seizures

After recovery from surgery, mice were individually housed in custom-fabricated plethysmography chambers and monitored 24 h a day. Plethysmography chambers were built to comply with requirements for continuous housing described in the Guide for the Care and Use of Laboratory Animals (Council, 2011). The floor of the chambers had approximate dimensions of 4.5 × 4.5 inches (>20 sq. inches) and 7 inches tall. There were ports for air in and air out, and for pressure monitoring. The chamber was supplied with a continuous flow of room air at approximately 400 ml/min via supply and exhaust air pumps (MK-1504 Aquarium Air Pump; AQUA Culture) balanced to maintain chamber pressure near atmospheric. Mice had access to a continuous supply of water and food. The surgically implanted headsets were attached to a custom low torque swivel cable, allowing mice to move freely in the chamber.

To assess breathing frequency, the pressure of the epilepsy monitoring unit chamber was measured with an analog pressure transducer (SDP1000-L05; Sensirion). ECoG and ECG signals were amplified at 2,000 and bandpass filtered between 0.3–100 Hz and 30–300 Hz, respectively, with an analog amplifier (Neurodata Model 12, Grass Instruments Co.). Biosignals were digitized with a Powerlab 16/35 and recorded using LabChart 7 software (AD Instruments, Inc.) at 1 kS/s. Video acquisition was performed by multiplexing four miniature night vision-enabled cameras and then digitizing the video feed with a Dazzle Video Capture Device (Corel, Inc.) and recording at 30 fps with LabChart 7 software in tandem with biosignals.

### Recording of ECoG, ECG, and Breathing for Audiogenic Seizures

For simultaneous ECoG, ECG, and breathing during audiogenic seizures, the same surgical procedure and experimental setup described above was used. Although mice remained in the chambers 24 hours/day, recording only took place during periods of audiogenic seizure stimulation. To induce audiogenic seizures, a 15 kHz signal was generated using Tone Generator software (NCH Software, Inc.), amplified using a Kinter K3118 stereo amplifier (Kinter United States), and converted to sound using a small 3-watt speaker lowered into the plethysmography chamber.

For recording of only breathing in pharmacological experiments, non-implanted mice were placed in the chambers immediately after injection of pharmacological agents. Stimulation of audiogenic seizures and recording of breathing were performed as described above.

### Breathing and Heart Rate Detection

Individual breaths and heart beats were identified as inspiratory deflections in the pressure transducer signal and R waves in the ECG signal, respectively, using Spike2 software (Cambridge Electronic Design, Ltd.). A breath was scored when the downward deflection went below a certain hysteresis value determined by the experimenter and rose back above a threshold of 0 mV. The minimum time between breaths was set to 0.05 s. Similarly, an R wave was identified when an upward deflection crossed a threshold value determined by the experimenter. The minimum time between R waves was set to 0.02 s. All breaths and R waves were inspected by the experimenter.

### Mechanical Ventilation

For the mechanical ventilation of P20-21 D/+ mice during an audiogenic seizure, we quickly placed a custom-made 3 mL pipet which fit snuggly over the mouse’s nose and mouth and provided regular pulses of air (∼1 mL at 2 Hz). The ventilation was terminated after the mouse initiated gasping behavior or 20 s after the animal experienced sudden death. A full video of this procedure is available (Supplementary Video 4).

### Adrenergic Receptor Pharmacology

All chemicals were purchased from Sigma Aldrich and were either of pharmaceutical grade or were sterile filtered prior to injection. Injections were given intraperitoneal in a volume of 50–100 μl sterile saline per mg of mouse weight (e.g., 0.1 mL for a 20 mg adult mouse). The concentration of each drug was based on the achieving the desired dosages of 2 mg/kg epinephrine HCl, 2 mg/kg norepinephrine HCl, and 3 mg/kg phenylephrine HCl, 10 mg/kg sotalol HCl, and 1 mg/kg prazosin HCl. Injection of 50–100 μl sterile saline per mg of mouse weight was used as control.

### Statistical Analysis

All interventions were conducted and analyzed with the experimenter blinded, except for mechanical ventilation. All data points denote biological replicates (i.e., no animal was used more than once for the same test), except for data comparing spontaneous and audiogenic seizures, which are technical replicates and the animal numbers are reported in the figure legend. All average data values are mean ± SEM. Statistics were computed using GraphPad Prism version 7 or later (GraphPad Software, Inc.) and comparisons were considered statistically detectable when *P* < 0.05. Differences between two groups were assessed by unpaired, two-tailed Student’s *t*-test when distributions passed the D’Agnostino-Pearson Omnibus normality test and Mann-Whitney non-parametric test when any distribution failed to pass the normality test. Differences between more than two groups were assessed by one-Way or two-Way ANOVA followed by Holm-Sidak’s or Sidak’s multiple comparison tests, respectively. For the few cases where residuals of one-Way ANOVAs failed the D’Agnostino-Pearson Omnibus normality test (*P* < 0.05), we used the Kruskal-Wallis non-parametric test followed by Dunn’s multiple comparisons test. Comparison of survival proportions was done using a one-sided Fisher’s exact test.

## Results

### Characterization of Audiogenic Seizure Behavior in D/+ Mice

**FIGURE 1:**
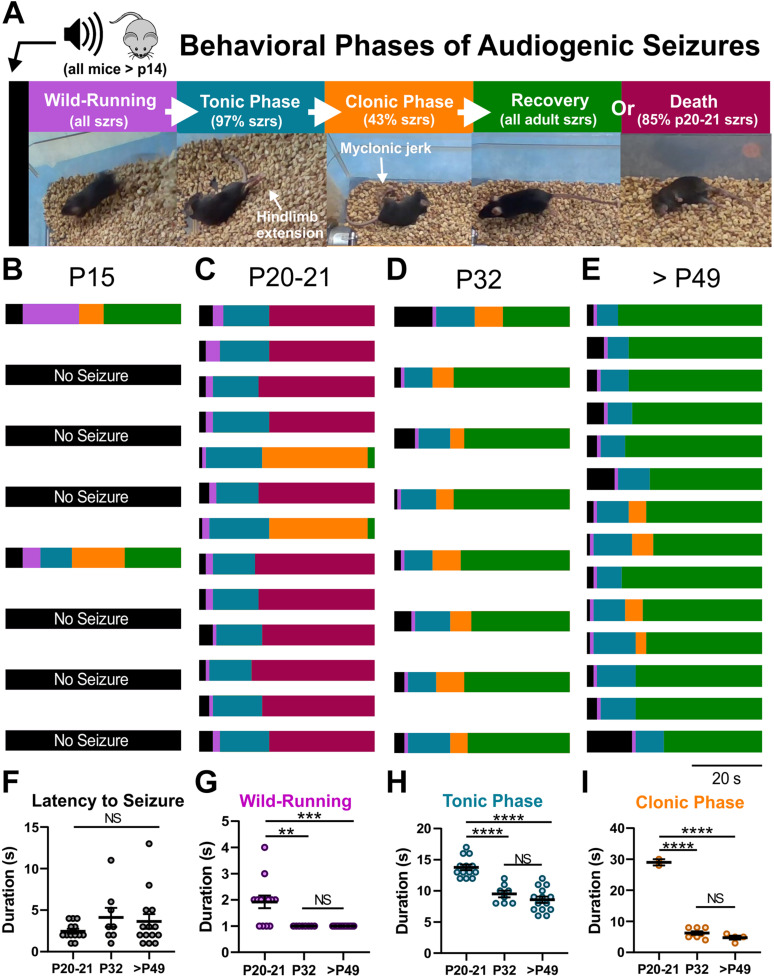
Audiogenic seizures and sudden death in D/+ mice. **(A)** In response to high-intensity acoustic stimulation, D/+ mice exhibit wild-running (purple), a tonic phase characterized by hindlimb extension (teal), a clonic stage (orange) characterized by rapid kicking of the hindlimbs, and recovery (green) as the animal rights itself and resumes normal movement throughout the cage, or sudden death (magenta). Audiogenic seizure behavioral progression in each mouse at developmental time points P15 **(B)**, P20-21 **(C)**, P32 **(D)**, and P49-P69 **(E)**. **(B)** Only 2 of 8 D/+ mice at P15 exhibited audiogenic seizures. **(C)** All 13 D/+ mice at P20-21 experienced audiogenic seizures, and 11 of 13 died directly following the tonic phase. The 2 that recovered did so after a relatively extended clonic phase. **(D)** At P32, all 8 D/+ mice had audiogenic seizures and recovered. **(E)** In adult D/+ mice (P49-P69), all mice experienced seizures followed by recovery. Duration of seizure phases, latency to seizure onset **(F)**, wild-running **(G)**, tonic phase **(H)**, and clonic phase **(I)** for D/+ mice ages P20-21 (*n* = 13), P32 (*n* = 8), and P49-P69 (14). **, ***, ****, and NS indicate *P* < 0.01, 0.001, 0.0001, >0.2, respectively, for *post hoc* comparison between age groups.

We serendipitously discovered that D/+ mice are susceptible to audiogenic seizures when a sonicator (Branson 200 Ultrasonic cleaner) was turned on in close proximity to D/+ mice. Further investigation using the same acoustic stimulus revealed that D/+ mice at ages P20-21, P32, and P49-P69 exhibit stereotyped seizure behaviors; wild-running followed by a tonic phase with hindlimb extension that was, in some cases, followed by a clonic phase consisting of myoclonic leg jerking (Figure 1A and Supplementary Video 1). Only 2 of 8 D/+ mice were sensitive to audiogenic seizures at P15 (Figure 1B), which we attribute to the fact that mice at this age are likely still developing their auditory system. Strikingly, all 13 D/+ mice tested at P20-21 experienced audiogenic seizures, 11 of which succumbed to sudden death immediately following the seizure (Figure 1C and Supplementary Video 2). In contrast, no deaths were observed in D/+ mice at P32 (*n* = 8) or P49-69 (*n* = 14) even though all mice experienced an audiogenic seizure (Figures 1D,E). Further characterization of the audiogenic seizures revealed that there were no differences in latency to seizure [*P* = 0.25; *F*_(2, 32)_ = 1.460; Figure 1F] although P20-21 D/+ mice experienced seizures with longer wild-running (^∗∗^*P* = 0.0017 and ^∗∗∗^*P* < 0.0002, respectively; Dunn’s multiple comparisons test; Figure 1G), tonic [^****^P < 0.0001 for both comparisons; *F*_(2, 32)_ = 32.99, Figure 1H], and clonic [^****^P < 0.0001 for both comparisons; *F*_(2, 32)_ = 211.5, Figure 1I] phases compared to D/+ mice of ages P32 and P49-69. No seizures were observed in WT littermates exposed to the same acoustic stimulus (*n* = 37 and Supplementary Video 3). It should be noted that due to the high incidence of death at P20-21, we only observed 2 instances of a clonic phase; thus, the statistical difference between ages in Figure 1I should be interpreted with appropriate caution.

We characterized the intensity-sensitivity of audiogenic seizures in adult D/+ mice using a custom speaker/microphone feedback system (see section “Materials and Methods”). To determine intensity-sensitivity, we exposed D/+ mice to pulses of ∼20 s, 14 kHz acoustic stimuli, increasing each consecutive pulse by ∼5 dB until an audiogenic seizure was triggered. All 5 D/+ mice had audiogenic seizures by 100 dB (Supplementary Figure 1).

Other mouse models that exhibit audiogenic seizures, as well as mice with different *Scn8a* mutations, have impaired hearing (Willott et al., 1995; Willott and Bross, 1996; Koay et al., 2002; Mackenzie et al., 2009; Heffner et al., 2019). Thus, we measured hearing sensitivity in D/+ and WT littermate control mice using auditory brainstem response (ABR) testing. Hearing thresholds at a range of frequencies (8, 11.3, 16, 22.4, and 32 kHz) were determined at 2 developmental time points; P56 and P112. We found mild hearing impairment at both ages (Supplementary Figure 2). At P56, ABR thresholds of D/+ mice were elevated compared to those of WT mice at 22.4 and 32 kHz [^∗^*P* = 0.0479 and 0.0322, respectively; *F*_(1, 18)_ = 3.929; Supplementary Figure 2C]. At P112, ABR thresholds observed in D/+ were elevated at 16 and 32 kHz and across the entire frequency range tested compared to WT mice [^∗∗^*P* = 0.0034, ^∗∗^*P* = 0.003, ^∗^*P* = 0.0156; *F*_(1, 9)_ = 8.844; Supplementary Figures 2D,F].

### Audiogenic and Spontaneous Seizures Have Similar Semiology

**FIGURE 2:**
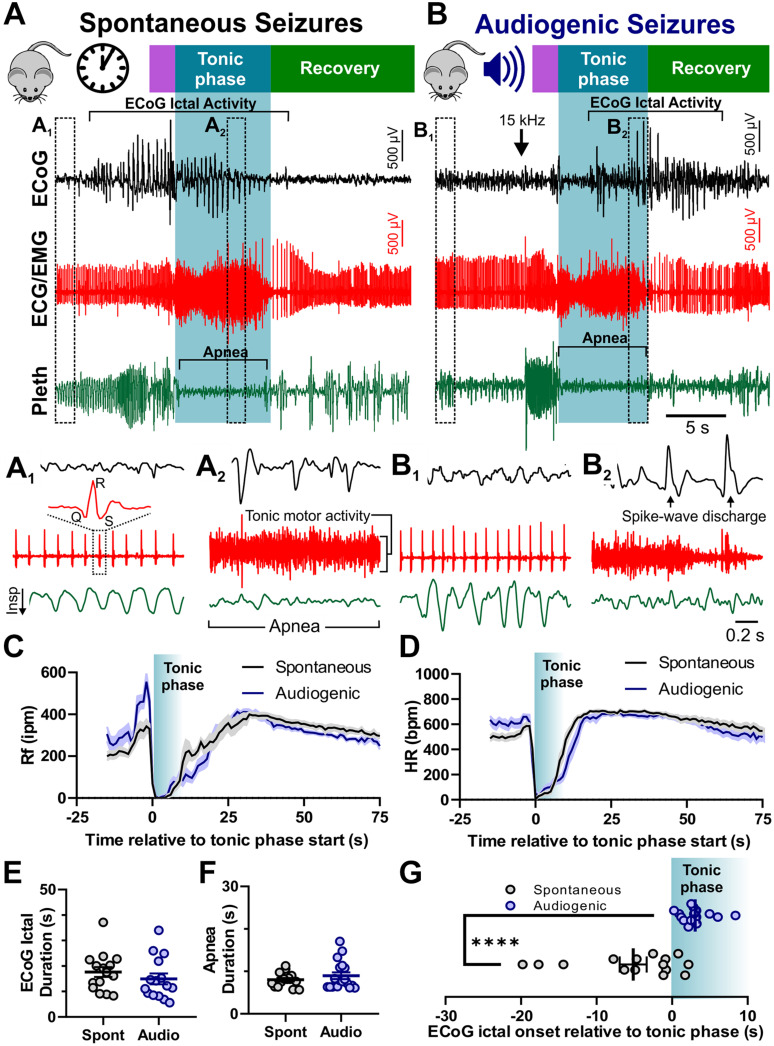
Audiogenic seizures resemble spontaneous seizures in D/+ mice. Example traces of electrocorticogram activity (ECoG), electrocardiogram activity (ECG/EMG), and breathing activity (Pleth) during example spontaneous **(A)** and audiogenic seizures **(B)**. Stages of seizure behavior are indicated above the traces: Wild-running (purple), tonic phase (blue), and recovery (green). ECoG ictal activity, bradycardia, and apnea are each highlighted in brackets. Arrow indicates onset of 15 kHz acoustic stimulation **(B)**. (A_1_,A_2_) Expanded data traces from regions indicated in **(A)**. (B_1_,B_2_) Expanded data traces from regions indicated in **(B)**. Note the high degree of tonic muscle activity and apnea in (A_2_,B_2_). **(C)** Average breathing frequency [Rf in inspirations per minute (Rf ipm)] binned every second during spontaneous (black, *n* = 16 seizures recorded from 4 mice) and audiogenic (blue, *n* = 15 seizures recorded from 7 mice) seizures. **(D)** Average heart rate in beats per minute (HR bpm) (HR) binned every second during spontaneous (black, *n* = 16 seizures recorded from 4 mice) and audiogenic (blue, *n* = 12 seizures recorded from 7 mice) seizures. **(E)** ECoG ictal duration for spontaneous (black, *n* = 15 seizures recorded from 4 mice) and audiogenic (blue, *n* = 15 seizures recorded from 6 mice) seizures. **(F)** Apnea duration for spontaneous (black, *n* = 16 seizures recorded from 4 mice) and audiogenic (blue, *n* = 18 seizures recorded from 7 mice) seizures. **(G)** Time of ictal onset relative to tonic phase. Audiogenic seizures (blue, *n* = 15 seizures recorded from 6 mice) exhibit delayed onset of ECoG ictal activity relative to tonic phase compared to spontaneous seizures (black, *n* = 15 seizures recorded from 4 mice) in D/+ mice. ^*⁣*⁣**^P < 0.0001 comparison between genotypes.

We recorded spontaneous and audiogenic seizures from adult D/+ mice with standard rodent video/Electrocorticogram (ECoG) techniques. In addition, we simultaneously recorded muscle/cardiac function and breathing frequency via electrocardiogram (ECG)/electromyogram (EMG) activity and plethysmography, respectively (Figure 2). Both spontaneous and audiogenic seizures present with a series of cortical spike-wave discharges in addition to a period noted by a large amount of tonic muscle activity, apnea, and bradycardia that coincides with the behavioral tonic phase (Figures 2A,B). We found no differences in ictal or postictal breathing (Figure 2C) or heart rate (Figure 2D), nor ECoG spike-wave discharge (Figure 2E) or apnea duration (Mann-Whitney Test; Figure 2F) between spontaneous and audiogenic seizures. We did observe a robust increase in respiratory frequency upon audio stimulation that was not observed in the spontaneous seizure type (Figure 2C, −5 to 0 s). Interestingly, we found that the onset of ECoG spike-wave discharge always occurred after the initiation of the tonic phase in audiogenic seizures, whereas it typically preceded the tonic phase in spontaneous seizures (^****^P = 0.0001; Mann-Whitney Test; Figure 2G). These studies suggest that audiogenic and spontaneous seizures in D/+ mice are comparable, sharing similar mechanisms.

### Audiogenic Seizure-Induced Death Is Due to Lack of Breathing Recovery After the Tonic Phase

**FIGURE 3:**
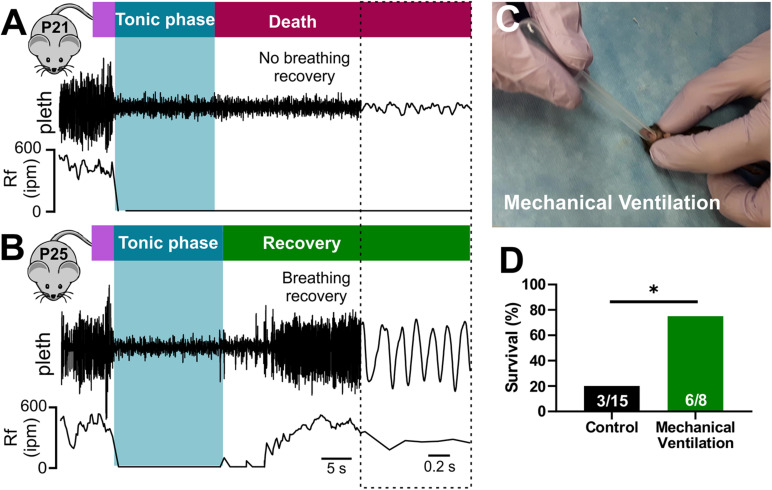
Seizure-induced respiratory arrest and sudden death in P20-21 D/*+* mice. Plethysmography recordings during audiogenic seizures in a P21 D/*+* mouse **(A)** and a P25 D/*+* mouse **(B)**. Behavioral seizure stages shown above traces. Wild running (purple) precedes a tonic phase (blue) followed by either death [red, **(A)**] or recovery [green, **(B)**]. **(A)** At the onset of the tonic phase of the audiogenic seizure, the P21 D/*+* mouse ceases breathing (Rf near zero) and never recovers. **(B)** In a P25 D/*+* mouse, breathing ceases during the tonic phase but recovers shortly after and the mouse survives. **(C)** Image of mechanical ventilation intervention to stimulate breathing after onset of an audiogenic seizure in P20-21 D/*+* mice. **(D)** Bar chart of survival of control non-ventilated (black, n = 15) and ventilated (green, n = 8) P20-21 D/*+* mice. Mechanical ventilation significantly improved rate of survival (*P < 0.05; one-sided Fisher’s exact test).

While both cardiac and respiratory arrest have been suggested as mechanisms of SUDEP, the primary mechanism of death remains debated (Massey et al., 2014; Devinsky et al., 2016). To examine breathing activity during terminal seizures, we conducted plethysmography recordings of P20-21 D/+ mice during audiogenic seizures (Figure 3). Audiogenic seizure-induced death observed in P20-21 D/+ mice occurred when apnea was initiated during the tonic phase and breathing failed to recover (Figure 3A). In mice only a few days older (P25), breathing resumed immediately after the tonic phase and all mice tested survived (*n* = 7; Figure 3B). This data supports the notion that at a critical time point of P20-21, a lack of breathing recovery causes seizure-induced death.

Stimulation of breathing via mechanical ventilation has been shown to rescue seizure-induced respiratory arrest in other mouse models of SUDEP (Faingold et al., 2010; Kim et al., 2018). Our novel ability to evoke seizures on command allowed us to assess whether this intervention could rescue the P20-21 D/+ mice. We performed mechanical ventilation (approximately 1 mL at 2 Hz delivered manually) to mice immediately after the onset of the tonic phase (Figure 3C and Supplementary Video 4). While only 3 of 15 non-ventilated mice survived, 6 of the 8 mice that were ventilated survived (^∗^*P* < 0.05; One-sided Fisher’s exact test; Figure 3D), demonstrating that respiratory arrest is the primary cause of seizure-induced death in P20-21 D/+ mice.

### Exogenous Adrenergic Receptor Stimulation Is Sufficient for Recovery Breathing and Survival of Audiogenic Seizures in Adult D/+ Mice

**FIGURE 4:**
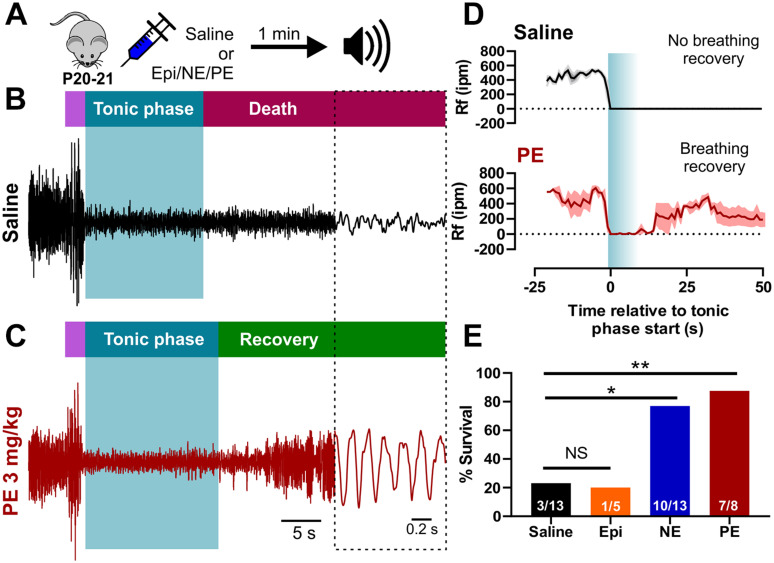
Acute activation of alpha-1 adrenergic receptors rescues seizure-induced sudden death in P20-21 D/+ mice. **(A)** P20-21 D/+ mice were injected (i.p.) with saline, 2 mg/kg epinephrine (Epi), 2 mg/kg norepinephrine (NE), or 3 mg/kg phenylephrine (PE) 1 min before stimulating an audiogenic seizure. Example plethysmography recordings of breathing during audiogenic seizures in P20-21 D/+ mice injected with saline control [black; **(B)**] or PE [red; **(C)**] 1 min before acoustic stimulation. In the saline-treated mouse, breathing ceased during the tonic phase and never recovered leading to death. In the PE-treated mouse, breathing also ceased during the tonic phase but recovered and the mouse survived. **(D)** Average breathing rates (Rf ipm) binned every second for saline-treated (black, *n* = 5) and PE-treated (*n* = 3, red) P20-21 D/+ mice. **(E)** Bar chart of survival of control saline-treated mice (black; *n* = 13), Epi-treated mice (orange; *n* = 5), NE-treated mice (blue; *n* = 13), and PE-treated mice (red; *n* = 8) reveals significantly elevated rates of survival in NE (*P < 0.05) and PE-treated (**P < 0.01) mice. Statistical comparisons made using a one-sided Fisher’s exact test.

Activation of adrenergic receptors has been shown to stimulate respiration in animals and humans (Whelan and Young, 1953; Zhang et al., 2017; Zhao et al., 2017). To determine if acute activation of adrenergic receptors could stimulate breathing and prevent audiogenic seizure-induced death, we injected P20-21 D/+ mice with either 2 mg/kg norepinephrine (NE) or an equal volume of sterile saline via intraperitoneal injection (i.p.) 1-min prior to audiogenic seizure stimulation (Figure 4A). Saline injection had no effect on survival and was not different from non-injected mice (*n* = 13; *P* = 0.6; One-sided Fisher’s exact test). In contrast, injection of NE resulted in breathing recovery and increased survival (10 of 13 mice) compared to saline injection (3 of 13 mice) (^∗^*P* < 0.05; One-sided Fisher’s exact test; Figure 4E and Supplementary Video 5). Despite relatively low sample size, 2 mg/kg epinephrine (Epi) rescued only 1 of the 5 mice indicating that it did not strongly improve survival as observed with NE (Figure 4E). Based on the fact that NE primarily stimulates the alpha adrenergic receptors (Motiejunaite et al., 2020), we decided to test whether selective alpha-1 receptor agonism might prevent seizure-induced death. Injection of 3 mg/kg phenylephrine (PE) promoted postictal breathing recovery and survival in 7 out of 8 mice (^∗∗^*P* < 0.01; One-sided Fisher’s exact test; Figures 4A–E and Supplementary Video 6), which suggests alpha-1 adrenergic receptor activity is sufficient to promote postictal recovery of breathing and survival of audiogenic seizures in P20-21 D/+ mice.

### Endogenous Adrenergic Receptor Function Is Required for Recovery Breathing and Survival of Audiogenic Seizures in Adult D/+ Mice

**FIGURE 5:**
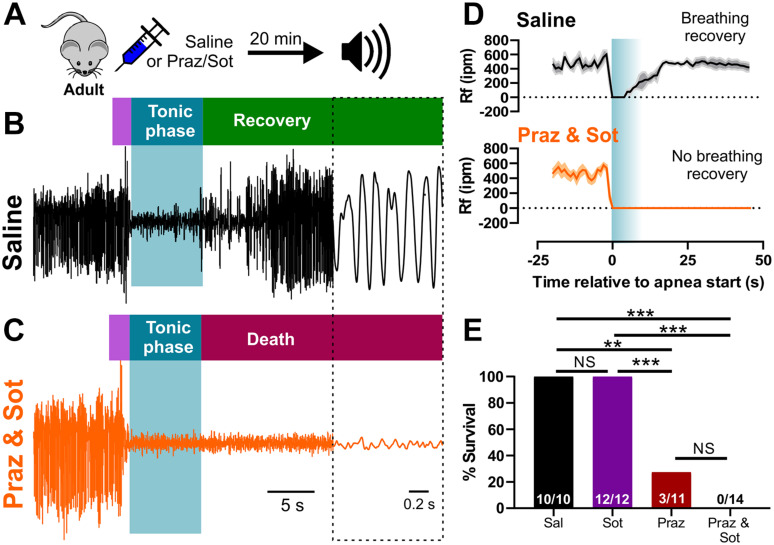
Inhibition of adrenergic receptors leads to seizure-induced respiratory arrest and sudden death in adult D/+ mice. **(A)** Adult D/+ mice were injected (i.p.) with saline, prazosin (1 mg/kg) and/or sotalol (10 mg/kg) 15 min before stimulation of an audiogenic seizure. Example plethysmography recordings of breathing during audiogenic seizures in D/+ mice treated with saline [black; **(B)**] or prazosin and sotalol [orange; **(C)**] ∼15 min before acoustic stimulation. Breathing recovers in the saline-treated adult mouse. However, breathing never recovers in the combined prazosin and sotalol-treated adult mouse after the audiogenic seizure. **(D)** Average breathing rates (Rf ipm) for saline-treated (black; *n* = 4) and prazosin/sotalol-treated mice (orange; *n* = 5). **(E)** Bar chart of survival of adult D/+ mice treated with saline (black; *n* = 10), sotalol (purple; *n* = 12), prazosin (red; *n* = 11), and prazosin/sotalol (no color; *n* = 14). Prazosin alone significantly reduced survival rate compared to saline injection (***P < 0.001; *n* = 11), with a similarly strong effect observed in mice treated with both prazosin and sotalol (***P < 0.001; *n* = 14). Statistical comparisons made using one-sided Fisher’s exact tests.

In order to test the necessity of adrenergic receptor function for breathing recovery and survival, we inhibited adrenergic receptor subtypes in adult D/+ mice which experience only non-fatal audiogenic seizures. We injected (i.p.) adult D/+ mice 15 min prior to audiogenic seizure stimulation with either 1 mg/kg prazosin (alpha-1 receptor antagonist), 10 mg/kg sotalol (beta receptor antagonist), a combination of the two, or saline as a control (Figure 5A). As expected, all 10 saline-injected adult D/+ mice had audiogenic seizures followed by recovery of breathing and survival (Figures 5B,D,E). We also observed that 12 out of 12 sotalol-treated mice survived, whereas only 3 of 11 prazosin-treated mice survived (^∗∗^*P* < 0.01 compared to saline-injected controls; ^∗∗∗^*P* < 0.001 compared to sotalol; Figure 5E and Supplementary Video 7). Interestingly, all 14 D/+ mice treated with the combination of prazosin and sotalol died immediately following an audiogenic seizure (^∗∗∗^*P* < 0.001; Figures 5C–E). Plethysmography recordings for prazosin/sotalol-treated adult D/+ mice revealed absence of breathing recovery (Figures 5C,D) similar to that of P20-21 D/+ mice (Figures 3A,B, 4A,B). These results suggest that activity of alpha-1 adrenergic receptors is required for the normal recovery of breathing and survival following audiogenic seizures of adult D/+ mice.

### Prevention of Seizure-Induced Death With Mechanical Ventilation Does Not Require Adrenergic Receptor Function

**FIGURE 6:**
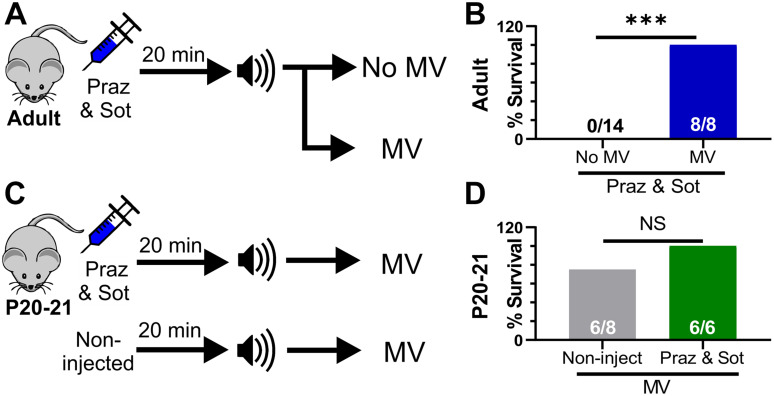
Mechanical ventilation does not require adrenergic receptor function to prevent seizure-induced sudden death. **(A)** Adult mice were injected (i.p.) with prazosin (1 mg/kg) and sotalol (10 mg/kg) 15 min before simulation of an audiogenic seizure. Some mice were either mechanical ventilated (MV) or not (No MV). **(B)** MV increased survival (8 of 8 adult D/+ mice) compared to No MV (***P < 0.001). **(C)** P20-21 D/+ mice were either non-injected or injected with a combination of prazosin (1 mg/kg) and sotalol (10 mg/kg) 15 min prior to stimulation of an audiogenic seizure. All mice received mechanical ventilation (MV). **(D)** 6 of 8 P20-21 D/+ mice that received MV only survived and were not statistically different from mice injected with a combination of prazosin (1 mg/kg) and sotalol (10 mg/kg) 15 min prior to stimulation of an audiogenic seizure that also received MV (6 of 6 mice). Statistical comparisons were made using one-sided Fisher’s exact tests.

Our results suggest that adrenergic receptor activity prevents death primarily via stimulation of breathing. We reasoned that even in the absence of functional adrenergic receptor signaling, any procedure to stimulate breathing would be able to rescue D/+ mice from seizure-induced death. To test this, we performed two sets of experiments: First, we injected (i.p.) mice with the combination of 10 mg/kg sotalol and 1 mg/kg prazosin 15 min prior to audiogenic seizure stimulation of adult D/+ mice, and either mechanically ventilated immediately after the start of the tonic phase or performed no intervention as a control (Figure 6A). Although the adrenergic antagonist cocktail caused audiogenic seizure-induced death in adult D/+ mice (0 out 14 survived; Figure 5E), all mice that received mechanical ventilation in addition to the adrenergic cocktail recovered breathing and survived (8 out of 8 survived; ^∗∗∗^*P* < 0.001; Figure 6B).

Second, we either injected (i.p.) P20-21 D/+ mice with the same adrenergic antagonist cocktail or gave no injection prior to audiogenic seizure-stimulation, and mechanically ventilated both groups immediately after the start of the tonic phase (Figure 6C). Even with relatively small sample size (*n* = 6 mice), adrenergic receptor blockade in P20-21 D/+ mice did not prevent the ability of mechanical ventilation to promote survival in any mice tested (6 of 6 survived; Figures 6C,D). This evidence supports the notion that adrenergic receptors function, likely indirectly, to stimulate breathing recovery after the tonic phase. However, any process that can directly stimulate breathing recovery will promote survival, even in the absence of adrenergic receptor function.

### Neither Suppressed Pre-ictal Breathing Rate nor Extended Tonic Phase Cause Seizure-Induced Death

**FIGURE 7:**
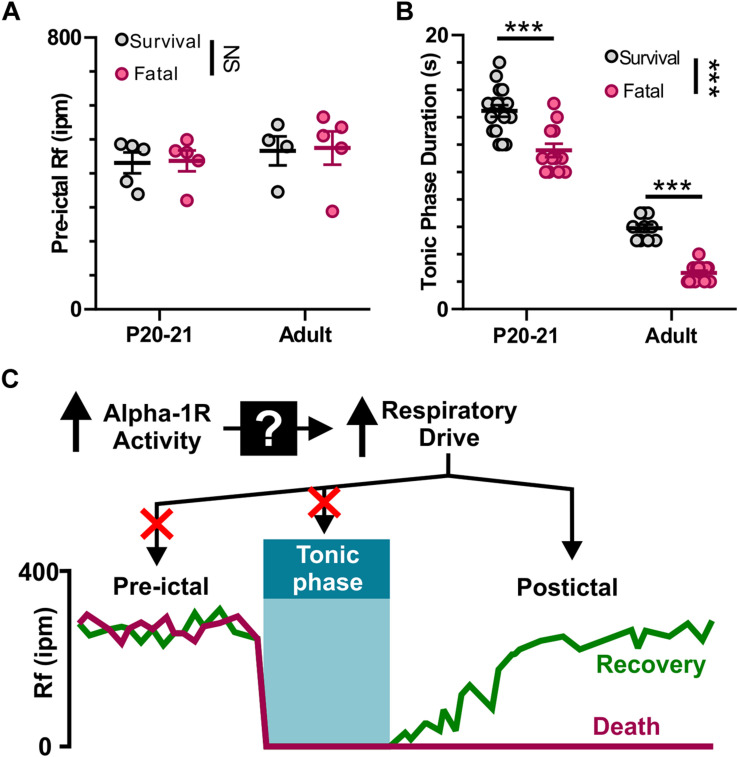
Neither pre-ictal breathing rate nor extended duration of tonic phase cause seizure-induced death. **(A)** Pre-ictal breathing rates in survival (gray) and fatal seizures (pink) in both P20-21 and adult D/*+* mice. **(B)** Tonic phase duration in P20-21 and adult D/*+* mice for both survival and fatal seizures. **(C)** Graphic summary: At the onset of tonic phase of seizures, breathing stops. Recovery of breathing from this apnea determines whether or not the animal recovers and survives. Mechanistically, alpha-1 receptors function upstream to drive respiration and prevent seizure-induced death. ***, NS indicate *P* < 0.001, and > 0.2, respectively, for main effect of survival vs. fatal and post hoc comparisons between age groups.

We found that apnea occurs during all audiogenic seizures in D/+ mice older than P15, and whether an individual seizure is fatal or not depends on recovery of breathing after this initial apnea. It is possible that adrenergic receptor activity promotes basal breathing, and this increases likelihood of survival. We found no difference in pre-ictal breathing rate between fatal and survival seizures [*F*_(1, 15)_ = 0.0351; *P* = 0.8539; Figure 7A]. It is also possible that a long tonic phase apnea could preclude the ability of respiratory neural circuitry to reinstate breathing. Contrary to this idea, we found that the behavioral tonic phase (i.e., duration of hindlimb extension) for fatal audiogenic seizures was actually shorter in duration than in mice that survived [*P* < 0.0001, *F*_(1, 49)_ = 30.80; Figure 7B]. Taken together, our studies suggest that the effect of adrenergic receptor-mediated seizure survival is due solely to increased respiratory drive immediately postictal (Figure 7C).

## Discussion

In this report, we present the novel finding that mice expressing the patient-derived N1768D *SCN8A* mutation (D/+) have audiogenic seizures that are nearly identical to spontaneous seizures. Furthermore, at the specific time point of P20-21, these audiogenic seizures result in sudden death, which can be rescued by mechanical ventilation immediately after the onset of an audiogenic seizure, indicating that breathing cessation is the primary cause of death. We report that blockade of adrenergic receptors with prazosin results in seizure-induced death in adult mice, while activation of adrenergic receptors with phenylephrine rescues seizure-induced death in P20-21 mice. These results indicate that adrenergic receptor activity, most likely the alpha-1 subtype, is critical for breathing recovery after tonic seizures and represents a potential therapeutic intervention for SUDEP.

### *SCN8A* Mutant Mice Have Audiogenic Seizures

*SCN8A* encephalopathy is a severe genetic epilepsy syndrome and neurodevelopmental disorder characterized by refractory seizures, cognitive and motor dysfunction, and a substantial risk for SUDEP (Veeramah et al., 2012; de Kovel et al., 2014; Estacion et al., 2014; Ohba et al., 2014; Blanchard et al., 2015; Larsen et al., 2015; Gardella et al., 2018; Zaman et al., 2019). *Scn8a* alleles containing patient-derived mutations form Na_*V*_1.6 voltage-gated sodium channels that create aberrant neuronal excitability in various cortical neurons including hippocampal CA1 (Lopez-Santiago et al., 2017; Baker et al., 2018; Bunton-Stasyshyn et al., 2019), entorhinal cortex (Ottolini et al., 2017), subiculum (Wengert et al., 2019), and layer V somatosensory cortex (Bunton-Stasyshyn et al., 2019). Our findings that *SCN8A* mice experience audiogenic seizures and seizure-induced sudden death suggest that additional regions, such as the inferior colliculus and amygdala which have been previously implicated in audiogenic seizures and sudden death (Wada et al., 1970; Millan et al., 1986; Faingold et al., 1992, 2017; Coffey et al., 1996; Dlouhy et al., 2015; Kommajosyula et al., 2017; Ribak, 2017; Marincovich et al., 2019), may also be functionally impacted by *SCN8A* mutations. In particular, prior studies have revealed intrinsic hyperexcitability in inferior colliculus neurons from rats susceptible to audiogenic seizures (Li et al., 1994). Interestingly, another study investigating a mouse expressing the R1627H *SCN8A* mutation found hearing impairment, abnormally elevated activity in inferior colliculus, and audiogenic seizures in response to acoustic stimulation (Makinson et al., 2016). Our results encourage future examination of various additional brain regions in models of *SCN8A* encephalopathy to more precisely identify the neuronal mechanisms responsible for the audiogenic seizures and sudden death.

Risk of death from audiogenic seizures in the D/+ mice was strongly age-dependent: Sudden death due to audiogenic seizure occurred with high probability in P20-21 mice, but was never observed in adult mice. This is in contrast to the mortality rate from spontaneous seizures in D/+ mice, where premature death can occur starting around 8 weeks of age and reaches 50% mortality by 1 year (Wagnon et al., 2015). The age-dependent differences in seizure phenotype and risk for sudden death are likely attributable to developmental changes in hearing, *SCN8A* expression, adrenergic receptor expression (possibly the alpha-1 subtype), as well as additional unknown factors. Gain-of-function mutations in *SCN8A* could lead to altered wiring of auditory neural circuits that favor initiation of audiogenic seizures. In support of this notion, mice harboring loss-of-function *SCN8A* mutations did not exhibit audiogenic seizures despite the presence of hearing impairment, thus it is unlikely that hearing impairment alone is sufficient for audiogenic seizures (Mackenzie et al., 2009). Additionally, endogenous alpha-1 adrenergic receptor function at P20-21 may be insufficient to rescue breathing and prevent seizure-induced sudden death. The fact that we do not observe spontaneous death at P20-21 in D/+ mice suggests that although they are capable of having audiogenic seizures at this age, they likely do not experience spontaneous seizures, consistent with previous reports (Wagnon et al., 2015). As to the low mortality of audiogenic seizures in adult D/+ mice, we have observed that D/+ mice have many non-fatal spontaneous seizures prior to death (unpublished observations). However, in the present study, we never induced audiogenic seizures more than three times in a single adult D/+ mouse. Thus, the likelihood of death from any single audiogenic vs. spontaneous seizure is likely not different between spontaneous and audiogenic seizures. Future studies as to how developmentally determined alpha-1 adrenergic signaling relates to seizure-induced sudden death are needed to further clarify the mechanism(s) of death in P20-21 D/+ mice. Our results demonstrating that norepinephrine and phenylephrine administration improves survival suggest that endogenous release of these alpha-1 adrenergic receptor-targeting monoamines at P20-21 is impaired in D/+ mice.

Similar to previous reports of other mouse models susceptible to audiogenic seizures, our recordings of auditory brainstem responses revealed that D/+ mice have impaired hearing (Willott et al., 1995; Willott and Bross, 1996; Koay et al., 2002; Mackenzie et al., 2009; Heffner et al., 2019). To date, there have been no reports of *SCN8A* encephalopathy patients that exhibit hearing abnormalities or audiogenic seizures. Further audiological evaluation of patients with *SCN8A* mutations is warranted to shed light on the importance of hearing impairment and seizures.

To our knowledge this is the first model of *SCN8A* encephalopathy which exhibits spontaneous seizures and the reliable induction of both non-fatal and fatal seizures. Since seizure-induced death can be reliably induced, our model allows for a more in-depth examination of the cascade of events that lead to either fatal or non-fatal seizures, increasing the versatility of this clinically relevant model of epilepsy. Importantly, audiogenic seizures highly resemble spontaneous seizures in D/+ mice with respect to ECoG activity, heart rate, and breathing. The differences observed in relative timing of ECoG ictal activity and onset of tonic phase might indicate different focal regions between audiogenic and spontaneous seizures. Nonetheless, our data support the notion that audiogenic seizures can be utilized as a model to understand mechanisms of seizure semiology and seizure-induced death.

### Respiratory Arrest Contributes to Seizure-Induced Death

There is increasing evidence that respiratory arrest is the primary cause of death in SUDEP. Most witnessed cases of SUDEP are preceded by convulsive seizures (Hesdorffer et al., 2011; Nashef et al., 2012; Ryvlin et al., 2013), and oxygen desaturation due to breathing complications is common during and after convulsive seizures (Bateman et al., 2008; Lacuey et al., 2018; Vilella et al.,2019a,b). SUDEP events where cardiorespiratory parameters are adequately recorded are understandably limited; however, in these few cases patients experienced respiratory arrest prior to terminal asystole (Ryvlin et al., 2013), suggesting the primacy of respiratory failure. Data obtained from mouse models of SUDEP support this notion. Death is due to respiratory arrest for the stimulated seizures of *Lmx1b*^*f/f/p*^ and DBA/1&2 mice (Faingold et al., 2010; Buchanan et al., 2014; Irizarry et al., 2020), and the spontaneous seizure-induced deaths in *Cacna1a*^*S*218L^ mice and *Scn1a*^*R*1407X^, a mouse model of Dravet Syndrome (Kim et al., 2018; Jansen et al., 2019; Loonen et al., 2019). Breathing dysfunction is also reported for seizures induced under urethane anesthesia using *Kcna1 KO*, *RyR2*^*R*176Q^, *Cacna1a*^*S*218L^ mice, and Sprague-Dawley rats (Aiba and Noebels, 2015; Aiba et al., 2016; Loonen et al., 2019).

It has been previously reported that D/+ mice have cardiac arrhythmias and experience bradycardia prior to death (Frasier et al., 2016); however, breathing was not recorded in these studies and the sequence of events during seizure-induced death was not presented. Due to the young age of the mice in our audiogenic model of seizure-induced death we did not record cardiac function. Thus, bradycardia, or other cardiac abnormalities, could still play a role in the mortality of D/+ mice. However, the observation that mechanical ventilation prevents seizure-induced death suggests that breathing cessation is clearly an important factor in death.

### Tonic Phase Apnea and Failure of Breathing Recovery

Many studies concerned with breathing cessation as a mechanism of SUDEP speak of seizure-induced respiratory arrest (S-IRA). Often, S-IRA is synonymous with seizure-induced death; drugs that prevent death also prevent S-IRA (Faingold et al., 2010; Zeng et al., 2015; Zhang et al., 2017; Irizarry et al., 2020). In D/+ mice, we find that S-IRA occurs during all audiogenic seizures and coincides with the behavioral and electrographic tonic phase, which we refer to as tonic phase apnea. D/+ mice only experienced seizure-induced death when breathing did not recover immediately after the tonic phase. This discrepancy could be attributed to the fact that for most audiogenic seizure-induce death experiments, respiratory activity is assessed by visualization, which could make it difficult to ascertain breathing, or the lack thereof, during convulsions. Another factor could be that tonic seizures in DBA1/2J mice, which are used in the majority of preclinical SUDEP research, almost always produce death, making S-IRA and death coincident with one another (Martin et al., 2020). Considering tonic seizures are associated with apnea in humans (Gastaut et al., 1963; Wyllie, 2015), it is likely that most, if not all, tonic seizures in mouse models produce apnea.

It is unclear whether tonic phase apnea is necessary for postictal apnea and death, as there is no method to selectively prevent the tonic phase from occurring. However, it has been shown that DBA1/2J and 129/SvTer mice die from tonic, but not clonic, seizures (Martin et al., 2020), implying the tonic phase is important and perhaps necessary for seizure-induced death. Thus, determining cellular and molecular underpinnings of both tonic phase apnea and failure of breathing recovery will be important foci for future SUDEP research.

### Adrenergic Signaling and Seizure Survival

Mechanisms of seizure-induced death are poorly understood. Many proposed mechanisms involve impaired brainstem neural activity occurring due to synaptic seizure spread or spreading depolarization that impairs function of respiratory centers in the medulla producing central (Aiba and Noebels, 2015; Aiba et al., 2016; Salam et al., 2017; Ellis et al., 2018; Jansen et al., 2019; Loonen et al., 2019) or obstructive (Weissbrod et al., 2011; Nakase et al., 2016; Villiere et al., 2017) apnea. Seizure spread to non-medullary sites, such as the amygdala also suppresses breathing and is proposed to cause breathing cessation (Dlouhy et al., 2015; Marincovich et al., 2019; Nobis et al., 2019).

A body of work demonstrates the impairment of neuromodulator systems and their utility to rescue death in mouse models of SUDEP (Faingold et al., 2011, 2016; Buchanan et al., 2014; Zeng et al., 2015; Devinsky et al., 2016; Zhan et al., 2016; Zhang et al., 2017; Zhao et al., 2017; Feng and Faingold, 2017; Ellis et al., 2018; Kruse et al., 2019). Much of this work centers on the serotonergic system. Chronic administration of agents that increase serotonin levels can reduce the incidence of seizure-induced death (Faingold et al., 2011; Zeng et al., 2015; Feng and Faingold, 2017). In addition, genetic lesion of serotonergic raphe neurons elevates susceptibility of death after maximal electroshock seizures (Zhan et al., 2016), and optogenetic stimulation of the dorsal raphe reduces occurrence of seizure-induced death in DBA1 mice (Zhang et al., 2018). Noradrenaline has recently been shown to have a similar effect (Zhang et al., 2017; Zhao et al., 2017), and it appears that the ability of selective serotonin reuptake inhibitors (SSRIs) to prevent seizure-induced death is dependent on functional adrenergic signaling (Kruse et al., 2019).

We also demonstrate that functional adrenergic signaling is necessary for survival of seizures in adult D/+ mice, and this was largely dependent on functional alpha-1 receptors, and perhaps to a lesser extent, beta receptors. In addition, we show that exogenous stimulation of adrenergic receptors, most likely of the alpha-1 variety, is sufficient to prevent seizure-induced death in P20-21 D/+ mice. We intentionally utilized relatively high doses of epinephrine, norepinephrine, and phenylephrine in this study (Im et al., 1998; Gehrmann et al., 2000; Fleming et al., 2013; Suita et al., 2015). Despite low sample size, our finding that epinephrine did not prevent sudden death was surprising, however, when taken together with phenylephrine’s ability to prevent death and prazosin’s ability to produce it, our results suggest a critical role for alpha-1 receptors in seizure survival, similar to findings using maximal electroshock seizure-induced death (Kruse et al., 2019). For this reason, we hypothesize that activation of alpha-1 adrenergic receptors is a general requirement for survival after convulsive seizures and that acute augmentation of alpha-1 receptors could also reduce risk of seizure-induced sudden death in other seizures models. Further investigation into the mechanism underlying this difference between epinephrine and either norepinephrine or phenylephrine is warranted.

Precisely how adrenergic receptors promote breathing recovery and survival is still unclear. Previous studies have shown that intracerebroventricular injection of the norepinephrine reuptake inhibitor atomoxetine or alpha-1 receptor agonist phenylephrine prevents death from the audiogenic seizures of DBA1 mice (Zhao et al., 2017) or maximal electroshock seizures (Kruse et al., 2019), respectively. This could implicate a role in arousal neural circuitry, as a major function of noradrenergic signaling in the central nervous system is to increase arousal state (Broese et al., 2012). In our experiments, norepinephrine and phenylephrine were given intraperitoneally. Considering monoamine analogs do not cross the blood brain barrier (Olesen, 1972; Hardebo and Owman, 1980), our data suggests that peripheral alpha-1 adrenergic receptor activity is responsible for rescuing breathing in D/+ mice. Peripheral stimulation of alpha-1 receptors has little effect on heart rate but does increase peripheral resistance, which is crucial for maintaining blood flow to the heart and brain during life-threatening conditions of hypoxia and hemorrhage (Bond, 1985; Schultz et al., 2007). It is feasible that in the already compromised state of a severe convulsive seizure, multiple systems would be needed to coordinate full recovery.

## Conclusion

Taken together, our results highlight the D/+ mouse model of *SCN8A* encephalopathy as a new useful model for mechanistic investigation of SUDEP. We demonstrated the utility of this model by providing new evidence that respiratory arrest is the primary cause of death, and that interventional approaches to stimulate breathing including the augmentation of adrenergic receptor activity might be valuable for preventing SUDEP.

## Data Availability Statement

The raw data supporting the conclusions of this article will be made available by the authors, without undue reservation.

## Ethics Statement

The animal study was reviewed and approved by the Animal Care and Use Committee at the University of Virginia.

## Author Contributions

EWe, IW, and MP designed the study. EWe, IW, EWa, PW, and RG performed the experiments. EWe, IW, EWa, J-BS, and MP wrote the manuscript. All authors contributed to the article and approved the submitted version.

## Conflict of Interest

The authors declare that the research was conducted in the absence of any commercial or financial relationships that could be construed as a potential conflict of interest.

## Supplementary Material

Supplementary Figure 1 Intensity-dependence of audiogenic seizures in D/+ mice. Cumulative probability of adult D/+ with audiogenic seizures relative to the intensity of acoustic stimulation at 14 kHz. All mice exhibited seizures by 100 dB.

Supplementary Figure 2 D/+ mice exhibit mild hearing impairment. **(A)** Example auditory brainstem response (ABR) traces for a WT mouse at P56 in response to a 32 kHz tone. ABR threshold is indicated in green at 35 dB. **(B)** D/+ ABR example traces indicate a threshold of 55 dB (green trace). **(C)** Average ABR thresholds for WT (black, *n* = 10) and D/+ (red, *n* = 10) mice at P56. D/+ mice had significantly elevated ABR thresholds at 22.4 and 32 kHz. **(D)** Example ABR traces for a WT mouse at P112 in response to 32 kHz tone. ABR threshold is indicated at 60 dB (green trace). **(E)** Example ABR traces for a D/+ mouse at P112 in response to a 32 kHz tone with the ABR threshold indicated at 70 dB. **(F)** Average ABR thresholds for WT (black, *n* = 6) and D/+ (red, *n* = 5) reveal significantly elevated ABR thresholds in D/+ mice. ^∗^*P* < 0.05.

Supplementary Video 1 Audiogenic seizure in an adult D/+ mouse.

Supplementary Video 2 Audiogenic seizure and sudden death in a p21 D/+ mouse.

Supplementary Video 3 Adult WT mice do not have audiogenic seizures.

Supplementary Video 4 Mechanical ventilation prevents seizure-induced death in P21 D/+ mouse.

Supplementary Video 5 Acute norepinephrine administration prevents seizure-induced death in P21 D/+ mouse.

Supplementary Video 6 Acute phenylephrine administration prevents seizure-induced death in P21 D/+ mouse.

Supplementary Video 7 Prazosin-administration induces seizure-induced sudden death in adult D/+ mouse.
