# Supplementary figures and images for: Adrenergic Mechanisms of Audiogenic Seizure-Induced Death in a Mouse Model of SCN8A Encephalopathy
Source: Front Neurosci. 2021 Mar 4;15:581048. doi: 10.3389/fnins.2021.581048 (PMC7982890; doi:10.3389/fnins.2021.581048)

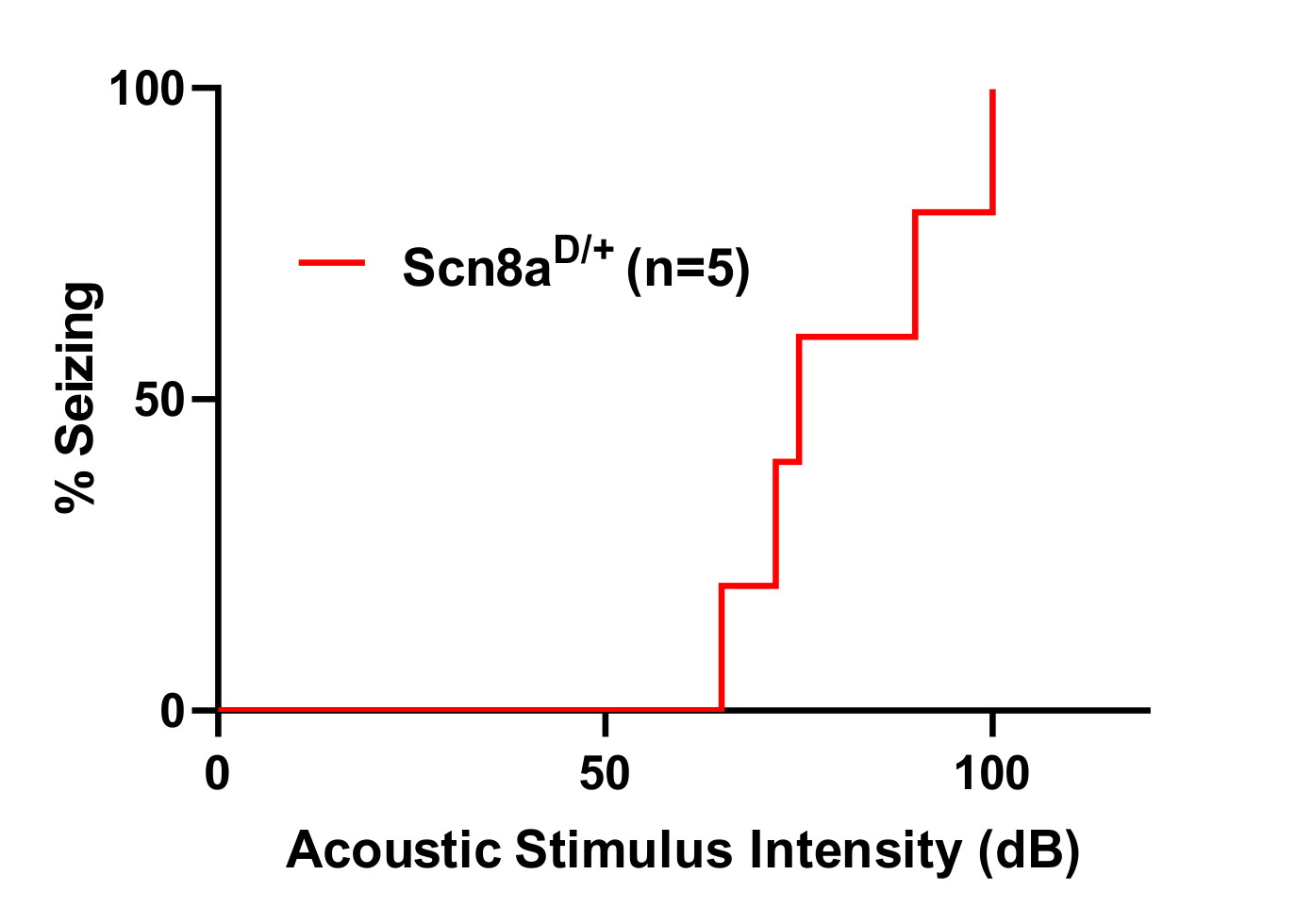

Supplement: Supplementary Figure 1 — Intensity-dependence of audiogenic seizures in D/+ mice. Cumulative probability of adult D/+ with audiogenic seizures relative to the intensity of acoustic stimulation at 14 kHz. All mice exhibited seizures by 100 dB. [file Image_1.TIF]

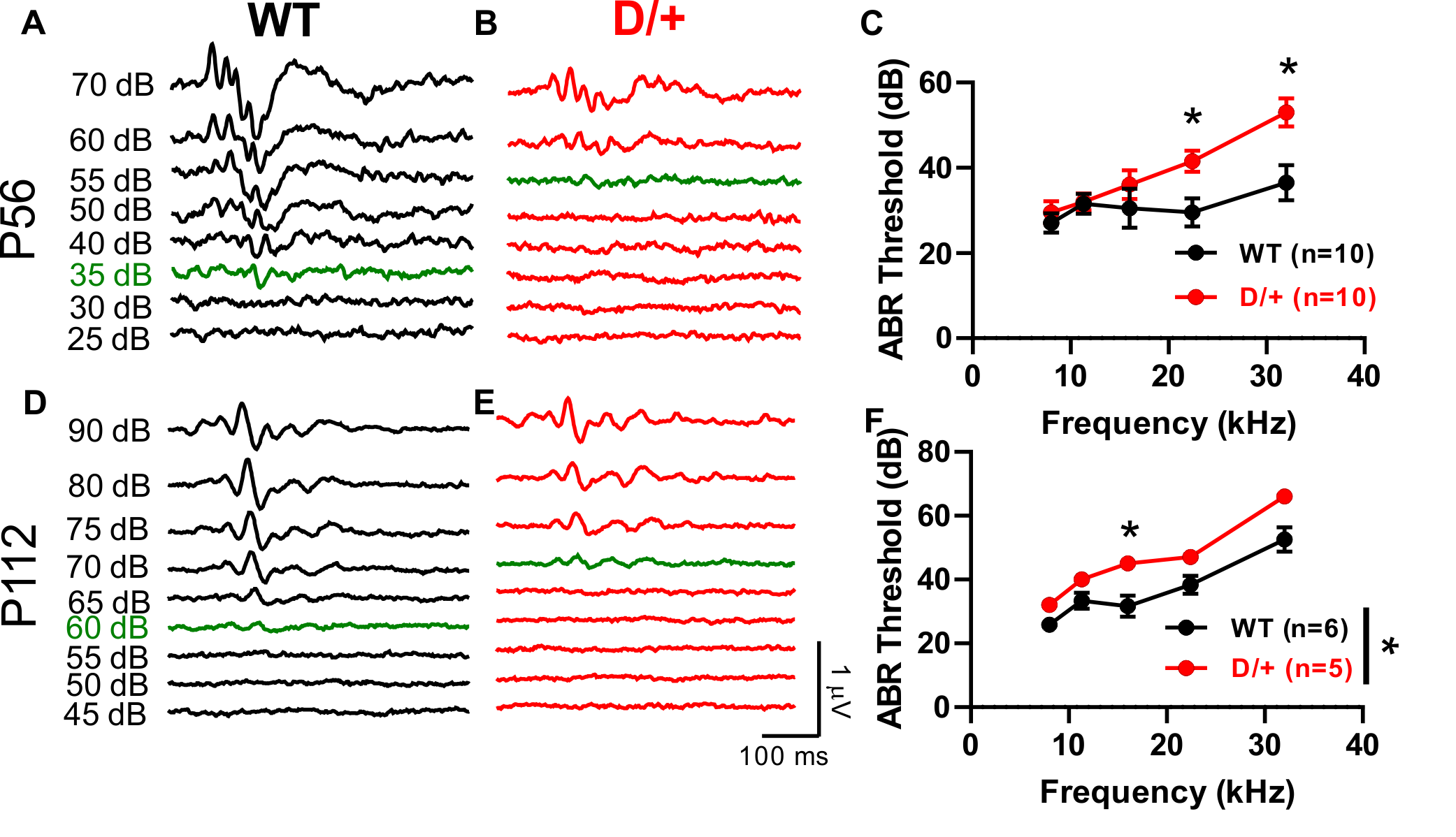

Supplement: Supplementary Figure 2 — D/+ mice exhibit mild hearing impairment. (A) Example auditory brainstem response (ABR) traces for a WT mouse at P56 in response to a 32 kHz tone. ABR threshold is indicated in green at 35 dB. (B) D/+ ABR example traces indicate a threshold of 55 dB (green trace). (C) Average ABR thresholds for WT (black, n = 10) and D/+ (red, n = 10) mice at P56. D/+ mice had significantly elevated ABR thresholds at 22.4 and 32 kHz. (D) Example ABR traces for a WT mouse at P112 in response to 32 kHz tone. ABR threshold is indicated at 60 dB (green trace). (E) Example ABR traces for a D/+ mouse at P112 in response to a 32 kHz tone with the ABR threshold indicated at 70 dB. (F) Average ABR thresholds for WT (black, n = 6) and D/+ (red, n = 5) reveal significantly elevated ABR thresholds in D/+ mice. ∗P < 0.05. [file Image_2.TIF]
